# Syntenic relationship of chromosomes in *Strongyloides* species and *Rhabditophanes diutinus* based on the chromosome-level genome assemblies

**DOI:** 10.1098/rstb.2022.0446

**Published:** 2024-01-15

**Authors:** Asuka Kounosu, Simo Sun, Yasunobu Maeda, Mehmet Dayi, Akemi Yoshida, Haruhiko Maruyama, Vicky Hunt, Asako Sugimoto, Taisei Kikuchi

**Affiliations:** ^1^ Department of Integrated Biosciences, Graduate School of Frontier Sciences, The University of Tokyo, Chiba 277-8562, Japan; ^2^ Department of Infectious Diseases, Faculty of Medicine, University of Miyazaki, 5200 Kihara, Miyazaki 889-1692, Japan; ^3^ Forestry Vocational School, Duzce University, 81620 Duzce, Türkiye; ^4^ Frontier Science Research Center, University of Miyazaki, Miyazaki 889-1692, Japan; ^5^ Department of Biology and Biochemistry, University of Bath, Bath BA27AY, UK; ^6^ Laboratory of Developmental Dynamics, Graduate School of Life Sciences, Tohoku University, Sendai 980-8577, Japan

**Keywords:** *Strongyloides ratti*, Hi-C, parthenogenesis, sex determination, sex chromosome evolution

## Abstract

The *Strongyloides* clade, to which the parasitic nematode genus *Strongyloides* belongs, contains taxa with diverse lifestyles, ranging from free-living to obligate vertebrate parasites. Reproductive strategies are also diverse in this group of nematodes, employing not only sexual reproduction but also parthenogenesis, making it an attractive group to study genome adaptation to specific conditions. An in-depth understanding of genome evolution, however, has been hampered by fragmented genome assemblies. In this study, we generated chromosome-level genome assemblies for two *Strongyloides* species and the outgroup species *Rhabditophanes diutinus* using long-read sequencing and high‐throughput chromosome conformation capture (Hi-C). Our synteny analyses revealed a clearer picture of chromosome evolution in this group, suggesting that a functional sex chromosome has been maintained throughout the group. We further investigated sex chromosome dynamics in the lifecycle of *Strongyloides ratti* and found that bivalent formation in oocytes appears to be important for male production in the mitotic parthenogenesis.

This article is part of the Theo Murphy meeting issue ‘*Strongyloides*: omics to worm-free populations’.

## 1. Introduction

**Figure 1.**
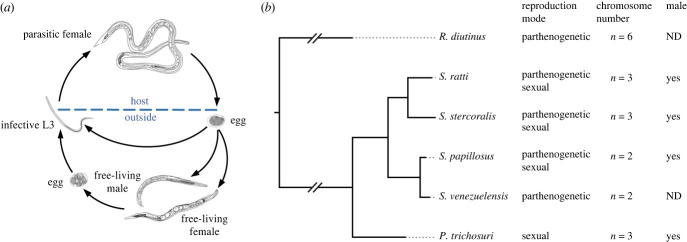
Diverse reproductive modes and karyotypes in the *Strongyloides* clade. (*a*) The lifecycle of *Strongyloides ratti*. The parasitic female (PF) parthenogenetically produces eggs in the host intestine that develop into free-living females (FLF), free-living males (FLM) or infective third-stage larvae (iL3). FLF and FLM sexually reproduce eggs that develop into iL3. iL3 penetrate the host's skin and develop into PFs in the host. (*b*) Phylogenetic relationship of species in the *Strongyloides clade*. Reproductive modes, karyotypes and presence of males are shown on the right side of the tree. *S. papillosus*, *Strongyloides papillosus*; *S. venezuelensis*, *Strongyloides venezuelensis*. ND, not detected.

*Strongyloides* species belong to nematode Clade IVa, which includes taxa with diverse lifestyles, including free-living forms (*Rhabditophanes*), facultative parasites of vertebrates (*Parastrongyloides*) and obligate parasites of vertebrates (*Strongyloides*), making the *Strongyloides clade* an attractive model for studying genome adaptation to parasitism. Reproductive strategies are also diverse in this group of nematodes. *Strongyloides* is almost unique in having alternating parasitic and free-living generations [1–3]. In the parasitic generation, there are only females and the adult female (parasitic female; PF) inhabiting the host's small intestine produces eggs by parthenogenesis. The eggs produced by the PF are excreted by the host in the faeces and undergo one of two developmental pathways: they develop directly into infective third-stage larvae (iL3) or they develop into a dioecious, sexually reproducing free-living male and female (FLM or FLF) whose offspring become exclusively iL3s. iL3s penetrate the host's skin and migrate to the gut, where they develop into parasitic adults [4] (figure 1*a*). *Parastrongyloides trichosuri* is similar to *Strongyloides* species, except that its parasitic generation is dioecious and that it can have unlimited cycles of its free-living adult generation [5,6]. The free-living nematode *Rhabditophanes diutinus*, which is phylogenetically placed at the ancestral position of *Strongyloides* and *Parastrongyloides*, reproduces only by meiotic parthenogenesis [7] (figure 1*b*). This suggests that they have changed their reproductive strategies as they have adapted to the parasitic lifestyle, and that changing reproductive strategies may be closely associated with parasitism. However, little is known about the mechanisms underlying the reproductive systems of this group of nematodes. In particular, it remains unclear how *Strongyloides* species are able to produce males by parthenogenesis (although parthenogenetic *R. diutinus* does not do this), and also how the offspring of free-living *Strongyloides* adults all develop into female iL3s by sexual reproduction.

Sex determination is classified according to the type of switch that triggers the sex determination cascade. Most animals use genetic sex determination (GSD), in which chromosomal differences such as sex chromosomes and autosomal genes are the main switches [8]. In some organisms, sex determination is controlled by the environment, such as temperature and sex ratio, and is known as environmental sex determination (ESD) [9,10], or can be determined randomly, known as stochastic sex determination [11].

In the phylum Nematoda, most species employ GSD that depends on sex chromosome (X chromosome) and the XO chromosome system (XX females/hermaphrodites and XO males) is considered to be the ancestral state of nematodes, although some filarial nematodes belonging to clade III have heteromorphic sex chromosomes (XX females and XY males) [12,13]. In *Caenorhabditis elegans,* whose sex determination is one of the best studied, the production of XO males from self-fertilized XX hermaphrodites involves the X chromosome nondisjunction and approximately 1% males are produced under the normal culture condition. By contrast, the proportion of male progeny from hermaphrodite and male mating is about 50%, which is the expected proportion. Multiple *C. elegans* mutants exhibiting 'Him' (high incidence of males) phenotype have been identified, in which up to approximately 50% of males are obtained from self-fertilized XX hermaphrodites due to chromosome non-disjunction during meiosis [14–16].

Sex determination in *Strongyloides* is considered to be GSD because previous karyotype observations in several *Strongyloides* species including *S. ratti* [17,18], *Strongyloides ransomi* [19] and *Strongyloides stercoralis* [20] found different numbers of chromosomes between the sexes. However, it remains unclear how the two types of karyotypes are generated by parthenogenesis in the PF, where meiotic processes are absent. In *S. papillosus*, DNA elimination, which removes a copy of the X chromosome region during embryonic cleavage divisions, has been observed and is suggested to be associated with male production [21,22]. In addition, changes in male ratio with external temperature and the time post-infection suggest the involvement of environmental effects [23,24].

The *Strongyloides* clade of nematodes have variable karyotypes. The ancestral species *R. diutinus* has been reported to have *n* = 5 karyotypes, whereas the sister taxa of *Strongyloides P. trichosuri* has *n* = 3. Karyotypes are also variable within the genus; *S. ratti* and *S. stercoralis* have *n* = 3, whereas *S. venezuelensis* and *S. papillosus* have *n* = 2, which is thought to be the result of a chromosome fusion event from the *n* = 3 ancestor. Recently, seven ancestral nematode chromosome elements called Nigon elements have been proposed and used to understand chromosome evolution in Rhabditida nematodes [13]. Although Nigon elements have generally been found as independent chromosomes throughout Rhabditida, including Clade III (*Brugia malayi*), Clade IV (*Bursaphelenchus* spp.) and Clade V (*Caenorhabditis* spp. and *Pristionchus* spp.), there are some exceptional groups that show mixed patterns of Nigon elements [13]. In particular, *S. ratti* showed a pattern of multiple breaks and fusions, exhibiting almost no conserved Nigon units in its three chromosomes [25].

We previously sequenced the genomes of four *Strongyloides* species (*S. ratti*, *S. stercoralis*, *S. papillosus* and *S. venezuelensis*) and two outgroup species *P. trichosuri* and *R. diutinus,* and used them for comparative analyses to identify gene families involved in parasitism [7]. Using these assemblies, we also performed analyses to investigate the evolution of genome structure, but these analyses were limited due to the fragmentary nature of the assemblies.

Here, we present chromosomally complete or nearly complete genome assemblies of *S. ratti*, *S. venezuelensis* and *R. diutinus* generated using long-read sequencing and high‐throughput chromosome conformation capture (Hi-C). We used the new assemblies to investigate the syntenic relationships of the chromosomes in this group and to gain a clearer view of the evolution of genome structure. In addition, the dynamics of sex chromosomes in the lifecycle of *S. ratti* were investigated using new techniques including single-worm sequencing and multiplexed FISH to elucidate the mechanisms of male production.

## 2. Material and methods

### (a) Parasite maintenances

*Strongyloides ratti* strain ED321 [26] and *S. venezuelensis* strain HH1 [27] were maintained in the Parasitology laboratory at the University of Miyazaki, Japan by serial passages in Wistar rats using a subcutaneous injection of iL3, as described in Hino *et.al.* [28]. *Rhabditophanes diutinus* KR3021 was maintained using OP50 bacteria as a food source on a Nematode growth Media (NGM) plate in the laboratory of parasite systems biology in the University of Tokyo.

### (b) DNA extraction, library preparation and sequencing

Approximately 30 000 iL3 were isolated from faecal culture and washed three times with phosphate buffered saline (PBS). They were transferred to 5 ml of Qiagen buffer G2 with 800 µg ml^−1^ proteinase K, 50 mM dithiothreitol (Wako) and 0.5 mg ml^−1^ RNase A (Invitrogen), and then incubated at 60°C for 2 h. Genomic DNA was spooled from ethanol precipitation following phenol–chloroform extraction and dissolved in 10 mM Tris (pH 8.0).

Nanopore sequencing libraries were prepared from 1 µg genomic DNA using the SQK-LSK109 sequencing Kit (Oxford Nanopore Technologies) according to the manufacturer's protocol. Libraries were sequenced with MinION R9.4.1 flow cells (Oxford Nanopore Technologies). The Nanopore reads were base called to generate FASTQ files using the Guppy v4.0.15 basecaller (Oxford Nanopore Technologies) with the supplied dna_r9.4.1_450bps_hac configuration and were quality-checked using NanoPlot v.1.31.0 [29]. Illumina paired-end sequencing libraries were prepared from 100 ng of DNA using the Nextera DNA FLEX Sample Prep kit and sequenced on HiSeq or MiSeq according to the manufacturer's protocol (Illumina).

The Hi-C libraries were prepared from around 100 000 fresh iL3 worms of *S. ratti* and *S. venezuelensis*, and from mixed-stage worms of *R. diutinus* using Arima-HiC + kit (Arima Genomics) and a Collibri ES DNA library prep kit (Thermo Fisher) according to the manufacturers' protocols. For *S. venezuelensis,* we additionally prepared parasitic female (PF) worms and prepared a PF HiC-library. The library was sequenced using a MiSeq instrument with the MiSeq reagent kit v.3 (101 cycles × 2). We obtained *ca.* 4.8 million, *ca*. 2.9 million and *ca*. 3.7 million paired-end reads for *S. ratti*, *S. venezuelensis* and *R. diutinus*, respectively. No discernible differences in the Hi-C contact map patterns were found between PF and iL3 worms of *S. venezuelensis* (electronic supplementary material, figure S1).

### (c) Genome assembly

The Nanopore long reads were assembled using NextDenovo v.2.4.0 (https://github.com/Nextomics/NextDenovo) with the parameters (genome size = 40 M and read_cutoff = 5k) for all of the species. After base correction by three rounds of Pilon v.1.23 [30] with the Illumina paired-end reads, the assembly was further scaffolded using the 3D-DNA pipeline v.180114 [31] without a misjoin correction process, and the Hi-C contact map was visualized using Juicebox v.1.11.08 [32].

### (d) Gene orthology and synteny analysis

Gene predictions for the new assemblies of *S. ratti*, *S. venezuelensis* and *R. diutinus* were performed with Braker v. 2.1.5 with RNAseq data of each species and a nematode protein set collected from WormBase as hints. To identify single-copy orthologues, we used OrthoFinder (v.2.5.4) [33] with *C. elegans*, *S. ratti*, *S. venezuelensis*, *S. stercoralis*, *S. papillosus*, *P. trichosuri* and *R. diutinus*. We employed TBtools [34] to show the positions of these orthologous genes on the chromosomes of each species.

### (e) Single worm sequencing

PFs were isolated from infected rats (8–10 dpi) as described in Suleiman *et al*. [35] and washed twice with pre-warmed (37°C) PBS. First-stage larvae (L1) were isolated from faeces of an infected rat (10–14 dpi) using the Baerman Fennel technique. To obtain free-living virgin females, males and iL3s (PF-iL3s), the L1 individual worms were cultured on 3% agar in a 12-well plate seeded with *E. coli* OP50 at 22°C for 2 days. To obtain iL3s developed from free-living eggs (FLF-iL3), free-living females and males (10 each) were transferred to a new 2% agar plate and incubated for at 22°C for 2 days. Single worms of each developmental stage were washed with PBS and transferred to 200 µl PCR tubes containing 10 µl worm lysis solution (10 mM Tris–HCl (pH 8.0), 500 mM EDTA, 500 mM NaCl, 800 µg ml^−1^ proteinase K (Qiagen), 50 mM dithiothreitol (Wako)). Samples were kept at −80°C until use.

DNA from single worms was extracted using Ampure beads (Beckman Coulter) and dissolved in 15 µl of nuclease-free water after incubation at 60°C for 1 h and 98°C for 10 min. Illumina sequencing libraries were prepared using Nextera DNA FLEX Sample Prep kit (Illumina) according to the manufacturer's protocol. Libraries were sequenced on Illumina HiSeq 2500. The Illumina short reads were aligned to the *S. ratti* genome assembly using SMALT v.0.7.6 [36] (https://www.sanger.ac.uk/science/tools/) and depth of coverage was calculated using mosdepth (v. 0.2.9). Coverage plots were plotted with ggplot2 in R studio (v. 4.2.1).

### (f) DAPI observation

PFs of *S. ratti* were isolated from rat small intestine (8–10 dpi) and washed twice with pre-warmed (37°C) PBS. Free-living females and males were isolated from faecal culture plates and washed three times with PBS. PF-eggs were isolated from PF using a 1 µm cell strainer (pluriSelect). FF-eggs were isolated from faecal culture plates. Worms pelleted in 1.5 ml microcentrifuge tubes (30–50 µl of packed worms per tubes) were immediately placed in liquid nitrogen for 1 min and kept at −80°C until use. Frozen worms were fixated in 500 µl of cold (−20°C) fixation buffer (acetic acid : methanol with 1 : 3) and vortexed for 1 min. Samples were then washed three times with PBS and resuspended in mounting media (Vectasheild; Vector Laboratories) with 5 µg ml^−1^ pf DAPI. Eggs isolated from PF or FF were pelleted in 1.5 ml microcentrifuge tubes, which were immediately placed in liquid nitrogen for 1 min and kept at −80°C before using. Frozen eggs were suspended with 20 µl of egg buffer (118 mM NaCl, 48 mM KCl, 2 mM CaCl_2_–2H_2_O, 2 mM MgCl_2_–6H_2_O and 25 mM Hepes) for 1 min. Eggs were placed on a Superfrost Plus Gold slide (Thermo Fisher Scientific) and covered with a coverslip. The slide glass was immediately placed on liquid nitrogen for 1 min. The coverslip was removed and the slide was placed in cold (at −20°C) fixation buffer (methanol and acetic acid 3:1) for 10 min. The excess of the fixation buffer was removed and replaced with mounting media (Vectasheild; Vector Laboratories) with 5 µg ml^−1^ of DAPI.

### (g) Multiplexed DNA fluorescence *in situ* hybridization (FISH)

Probes for multiplexed FISH for *S. ratti* chromosomes were designed using OligoMiner pipeline and prepared as described previously [37]. A total of 23 096 probes (10 404 for Chr1, 7277 for Chr2, 1929 for ChrX1 and 1486 for ChrX2; electronic supplementary material, table S2) were synthesized using a custom array oligo pool (GenScript, Japan). Probes were amplified using 5′ and 3′ specific primers with KAPA Taq Ready Mix mixture (2X) (Kapa Biosystems). Single strand RNAs (ssRNA) were synthesized using HiScribe T7 High Yield RNA Synthesis Kit (NEB) using 500 ng of amplified DNA as templates. Then, ssRNAs were transcribed into ssDNAs using Maxima H minus Reverse Transcriptase (Thermo Fisher Scientific) and ssRNA were removed via alkaline hydrolysis. ssDNA was purified with MinElute 96UF Plate (Qiagen).

FISH labelling was performed as described by Beliveau *et al.* [37]. Approximately 100 worms were pelleted by centrifugation, washed twice with PBS and then placed in liquid nitrogen for 1 min. The frozen worm-pellets were resuspended in cold fixation buffer (−20°C pre-cold acetic acid/methanol in proportions of 3 to 1), vortexed for 1 min and incubated for 10 min at −20°C with rocking. This fixation was repeated at room temperature with a fresh buffer. Worms were pelleted by centrifugation and washed twice in 2X SSCT (10X SSC diluted to 1X in H2O, 0.5% Triton X-100). Worms were resuspended in pre-warmed (37°C) 50% formamide 2X SSCT solution and incubated at 37°C for 6 h. Hybridization was performed with hybridization mixture (12% dextran sulfate, 20X SSC, 60% formamide, 100 pmol of primary probe per chromosome) at 37°C for 16 h. Worms were washed three times in pre-warmed (37°C) 2X SSCT, and hybridization of bridge oligos was performed in 60 ul of a mixture (2X SSC, 30% formamide, 100 pmol of bridge oligo and 100 pmol of each detection oligo). Worms were washed three times in 2X SSCT and resuspended in mounting media (Vectasheild; Vector Laboratories) with 5 µg ml^−1^ of DAPI.

### (h) Microscopy

Fluorescent microscopic observation was performed using BZ-X800 (KEYENCE). All image processing were done using the BZ-X800 imaging software (KEYENCE).

### (i) Digital PCR reaction

Primers to amplify approximately 100 bp regions located on Chr I, II, X1 and X2 were designed by Primer3Plus (v. 3.3.0) (electronic supplementary material, table S3). dPCR assays were performed in duplicates using a QIAcuity Digital PCR (dPCR) System and Nanoplate 8.5k 96-well plate (QIAGEN) under the following conditions: 95°C for 2 min, followed by 40 cycles of 95°C for 15 s, 55°C for 15 s and 72°C for 15 s and final extension at 40°C for 5 min. Each reaction mixture contained 5 µl of EvaGreen Master Mix (3X) (QIAGEN), 4 pmol each forward and reverse primers, 1.0 µl of DNA template and 3.5 µl of nuclease-free water.

## 3. Results

### (a) Chromosome-level genome assemblies of *S. ratti*, *S. venezuelensis* and *R. diutinus*

**Figure 2.**
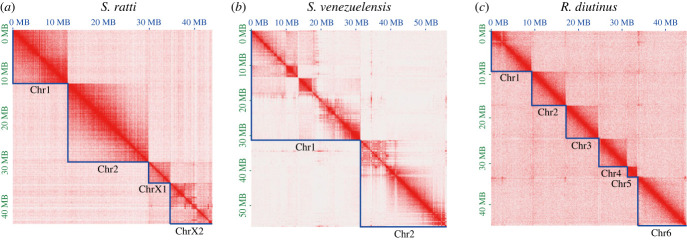
Hi-C chromosome contact maps of assemblies of (*a*) *S. ratti*, (*b*) *S. venezuelensis* and (*c*) *R. diutinus.* Each point represents a binned, normalized intensity of chromosomal contact measured by the number of ligated fragments sequenced. The darker colour of a block indicates higher contact intensity. The *x* and *y* axes indicate the cumulative length in millions of base pairs (Mb). The blue lines indicate HiC-based scaffolds corresponding to chromosomes. In the *S. ratti* Hi-C map, the x chromosome has been further subdivided into the ChrX1 and ChrX2 sub-clusters.

To obtain highly contiguous genome assemblies for detailed chromosome analyses in the *Strongyloides clade*, we re-sequenced and assembled the genomes of *S. ratti*, *S. venezuelensis* and *R. diutinus* using a long-read sequencer. By scaffolding these initial assemblies with Hi-C reads, we obtained chromosome-level genome assemblies for the three species. The previous genome assembly of *S. ratti* was already contiguous (12 large scaffolds assigned to the three chromosomes and 123 small unassigned scaffolds). In the present study, we obtained a further improved assembly consisting of four large scaffolds with an assembly size comparable to the previous one (43.1 Mb versus 43.8 Mb; table 1). In the *S. ratti* Hi-C contact map, two clear clusters representing chromosome I (Chr1) and chromosome II (Chr2) were observed, while the third Hi-C cluster was composed of two sub-clusters that we named ChrX1 and ChrX2 (figure 2*a*). The new genome assembly of *S. venezuelensis* is 44.3 Mb in length and clustered into two scaffolds, although the Hi-C contact signals were weak compared to the other nematodes analysed previously (figure 2*b*; electronic supplementary material, figure S1) [38–40]. *Rhabditophanes diutinus* was previously reported to have *n* = 5 according to the cytological observation [7], but our new assembly resulted in six distinct Hi-C scaffolds with five medium (6.5 Mb–11.2 Mb) and one small (approx. 2 Mb) scaffolds (figure 2*c*). By careful DAPI observation of *R. diutinus* eggs, we almost always identified six condensed chromosomes (CC; electronic supplementary material, figure S2), suggesting that the karyotype of *R. diutinus* is *n* = 6. However, when observing the oocytes with DAPI, we found a 6th small focus only in approximately 50% of the nuclei observed (electronic supplementary material, figure S2), which may be a reason why *n* = 5 was previously reported. Nevertheless, considering the exceptionally small size and the abnormal appearance of the oocytes, the sixth chromosome of *R. diutinus* may have a special character. In summary, this study greatly improved the assembly contiguities from the previous versions for all three species, taking them up to chromosome level (table 1).

**Table 1.** Genomic statistics of the *Strongyloides* spp. and *R. diutinus*.

	*S. ratti*	*S. venezuelensis*	*S. stercoralis*	*R. diutinus*
chromosome number	*n* = 3	*n* = 2	*n* = 3	*n* = 6
strain name	ED321	HH1C2	PV001	KR3021
assembly version	v.7	v.4	GCA_029582065.1	v.2
assembly size (Mbp)	43.9	56.0	44.4	44.8
number of genes	12 451	16 889	11 155	14 331
number of scaffolds	4	2	8	6
length of scaffolds	17.7 Mb	24.7 Mb	16.7 Mb	11.2 Mb
	12.1 Mb	31.3 Mb	11.6 Mb	9.3 Mb
	9.2 Mb		5.4 Mb	7.8 Mb
	4.7 Mb		5.1 Mb	7.5 Mb
			3.2 Mb	6.5 Mb
			1.1 Mb	2.3 Mb
			0.7 Mb	
			0.1 Mb	
GC content (%)	21.46	24.97	22.2	32.16
BUSCO % (proteins) complete/duplicated	80.0/4.7	76.7/5.0	77.7/4.4	76.6/6.8
BUSCO % (genome) complete/duplicated	78.0/2.1	74.8/1.9	78.5/3.1	72.9/3.4

### (b) Syntenic relationship of chromosomes in the *Strongyloides* clade

To investigate chromosome evolution in the *Strongyloides* clade, we performed synteny analysis using the newly generated chromosome-level assemblies of the three species with a *S. stercoralis* genome assembly downloaded from NCBI (accession number GCA_029582065.1). We identified 4694 one-to-one orthologues for the three *Strongyloides* species and *R. diutinus* and used them to reveal syntenic relationship of chromosomes in this group.

**Figure 3.**
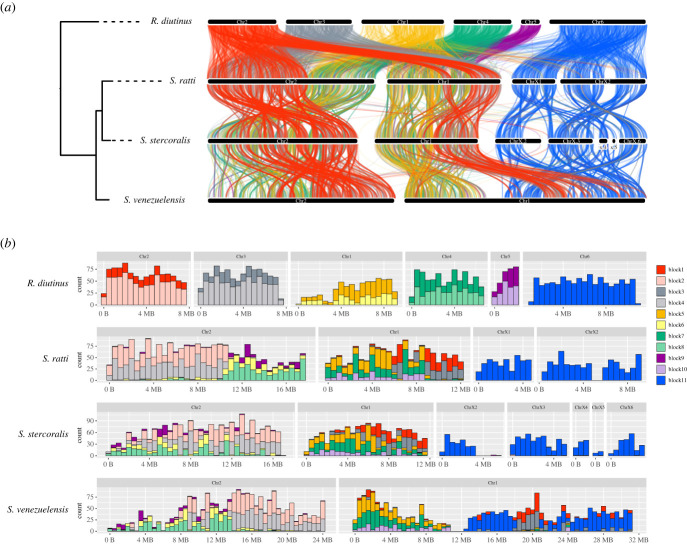
Relationship of chromosomes of *S. ratti*, *S. stercoralis*, *S. venezuelensis* and *R. diutinus*. (*a*) Syntenic relationship of the chromosomes of *S. ratti*, *S. stercoralis*, *S. venezuelensis* and *R. diutinus*. A total of 4969 one-to-one orthologues were used for the synteny analysis. Each horizontal black box represents a chromosome/scaffold. The loci of one-to-one orthologous genes in the four species are connected by lines coloured based on *R. diutinus* chromosomes. (*b*) Painting of chromosomes by *Strongyloides*-specific linkage blocks (*Strongyloides* blocks) in the *Strongyloides* clade. Eleven *Strongyloides* blocks were defined from the syntenic relationship between *R. diutinus* and *S. ratti*. Each plot shows the number of loci mapped in 0.5 Mb windows along a chromosome as a stacked histogram coloured by the *Strongyloides* blocks. The *x*-axis represents chromosome length and the *y*-axis represents block-defining loci per interval in each species.

Comparison of *S. ratti* and *S. stercoralis*, both of which have three chromosomes, revealed frequent intra-chromosomal rearrangements although inter-chromosomal rearrangements are very rare. *Strongyloides venezuelensis* has two chromosomes; one is syntenic to *S. ratti* Chr2 and the other appears to be the result of a fusion of two chromosomes (those syntenic to *S. ratti* Chr1 and ChrX). The comparison of *S. ratti* and *R. diutinus* revealed that Chr6 of *R. diutinus* is syntenic to *S. ratti* ChrX and the other five chromosomes (Chr1–Chr5) correspond to *S. ratti* Chr1 and Chr2. Chr1 to Chr5 of *R. diutinus* can be divided into two *S. ratti* syntenic groups; Chr2 and Chr3 of *R. diutinus* were mixed up and correspond to *S. ratti* Chr1 right hand and Chr2 left hand, whereas *R. diutinus* Chr1, 4 and 5 correspond to *S. ratti* Chr2 right hand and Chr1 left hand (figure 3*a*). Nigon elements, proposed as seven ancestral chromosome elements in rhabditine nematodes, have been used to reconstruct evolutionary history in other nematodes [41]. However, the Nigon elements are not conserved in *S. ratti* [25], which means that they are not useful for investigating chromosomal relationships in the *Strongyloides* clade. Therefore, we introduced 11 *Strongyloides*-specific linkage blocks (*Strongyloides* blocks) based on the comparison results of *S. ratti* and *R. diutinus* (figure 3*b*). Using these 11 blocks, each of the five *R. diutinus* autosomes can be explained as being the result of a fusion of two blocks. Chr2 of *S. ratti* can be explained as the result of multiple fusion events of different ages: an older fusion event of blocks 2/4 and blocks 6/8/9, and a relatively recent fusion of these fused chromosomes. A similar scenario can be applied to Chr1 of *S. ratti*, Chr1 and Chr2 of *S. stercoralis*, although *S. stercoralis* showed more extensive intrachromosomal rearrangements. As mentioned above, from the synteny plot we speculated that *S. venezuelensis* Chr1 is the result of a fusion of two chromosomes syntenic to *S. ratti* Chr1 and ChrX, followed by intra-chromosomal rearrangements that inserted a part of the autosomal region into the ChrX region. However, our linkage block analysis suggests that *S. venezuelensis* Chr1 is more likely to be the result of a fusion of three ancestral chromosomes: one with blocks 1 and 3, one with blocks 5, 7 and 10, and the block 11 corresponding to *S. ratti* ChrX.

In summary, chromosomes in the *Strongyloides* clade are likely to be the result of multiple chromosome fusion events and extensive intra-chromosomal rearrangements, especially in the case of autosomes. By contrast, across all genomes analysed, those regions syntenic to *S. ratti* ChrX were less mixed up with other chromosomes, suggesting long-term conservation of the X chromosome as seen in other animal groups [42].

### (c) X chromosome dynamics in *S. ratti* lifecycle

**Figure 4.**
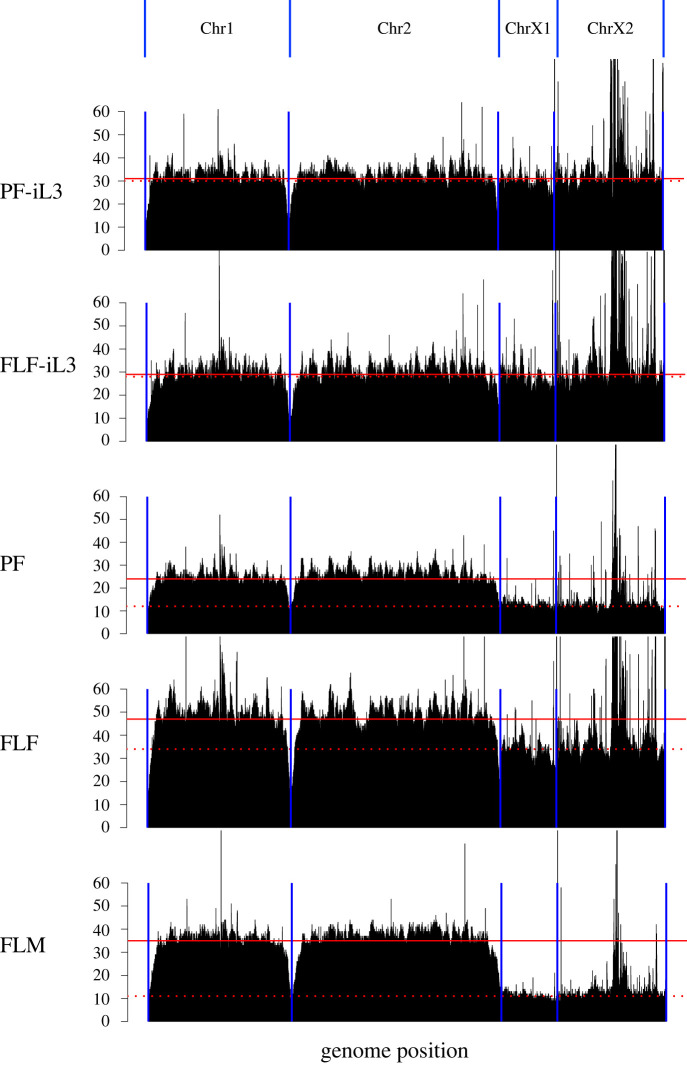
Read depth plots by single worm sequencing of five developmental stages of *S. ratti*. A representative sample out of 7–10 replicates for each developmental stage is shown in the figure. Median read depths of the 10 kb window are shown by the black bar for iL3s (PF-iL3 and FLF-iL3), parasitic female (PF), free-living female (FLF) and free-living male (FLM) *S. ratti*. The horizontal axis represents the physical position of each chromosome and the blue lines indicate the beginning and end of the chromosomes. Red straight and dashed lines represent the mean depth for autosomes (Chr1 and Chr2) and sex chromosomes (ChrX1 and ChrX2), respectively.

**Figure 5.**
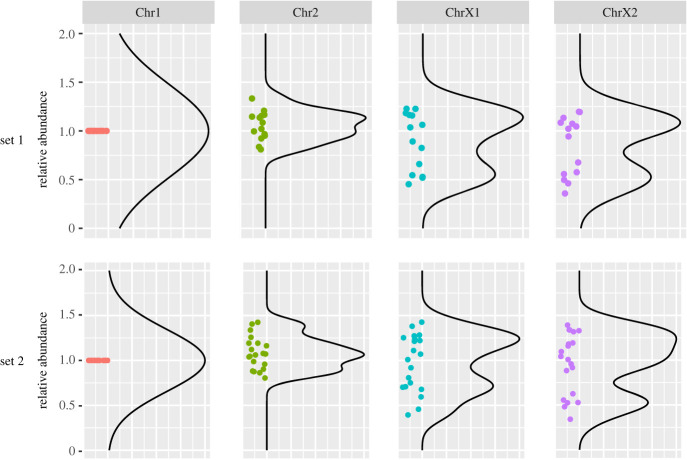
Copy number comparison of autosomes and X-chromosome in L1 larvae of *S. ratti*. The relative abundance of each chromosome in 13 (set 1) and 19 (set 2) individual L1 larvae was quantified by digital PCR (dPCR) using chromosome-specific primer pairs. Dots represent individual larvae. The values of Chr2, ChrX1 and ChrX2 were normalized to the Chr1 value. Black lines represent smooth lines generated with the stat_density function in R.

Next, we sought to investigate how the X chromosome contributes to sex determination in the *Strongyloides clade*. Using *S. ratti* as a model, we first compared the relative amount of the X chromosome to the autosomes by ‘single worm sequencing’ of five developmental stages including PF, FLF, FLM, iL3 developed directly from the PF egg (PF-iL3) and iL3 developed by mating of FLF and FLM (FL-iL3). Note that iL3s are reported to be always female. Sequence reads from individual worms were aligned to the new *S. ratti* assembly and depth of coverage was calculated using a 10-kb window (figure 4). The depth of coverage of iL3 showed a flat pattern, although peaks were observed in the plots, probably due to repetitive sequences in the genome. Similar patterns were observed in all iL3 samples regardless of PF-iL3 or FF-iL3 (electronic supplementary material, table S1), suggesting that the two iL3s have qualitatively the same genomes and are all females. By contrast, we found that the depth of ChrX1 and ChrX2 was reduced in the FLM. The depth of these regions showed 27% to 40% of the other two chromosomes in all FLM worms tested, although half would be expected if they used an XX/XO sex determination system. In addition, we also observed slight decreases in the ChrX1 and ChrX2 regions in PF and FF. The depths of these regions relative to the other chromosomes were 47–82% and 48–84% in PF and FF, respectively (electronic supplementary material, table S1). Then, to see the amount of X chromosome in L1 stage nematodes, we performed quantitative PCR using a dPCR system. We found that the copy numbers of ChrX1 and ChrX2 regions in iL3 were not significantly different from those of Chr1 and Chr2 regions. By contrast, the copy numbers of ChrX1 and ChrX2 regions in L1 showed two peaks in the distribution plot; one comparable to the autosomes and the other at about half of the autosome, indicating that L1 larvae may have two types of ChrX amounts, corresponding to XX and XO, respectively (figure 5). In conclusion, the amount of X chromosomes is smaller in males compared to females, although X chromosome reduction and/or autosome amplification seems to occur in all adult stages.

### (d) Chromosome observation by DAPI and multiplexed FISH in the PF oocytes of *S. ratti*

**Figure 6.**
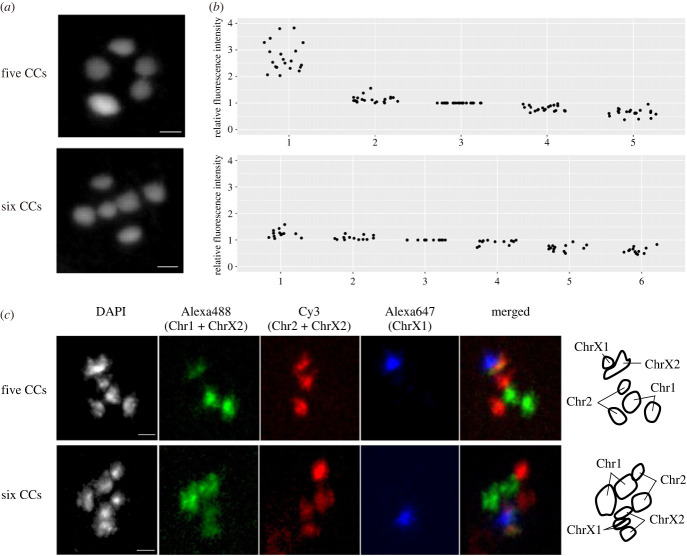
Microscopic view of nuclei in *S. ratti* parasitic female (PF) oocyte by DAPI staining and multiplex FISH. (*a*) Images of DAPI-stained nuclei with five or six foci in the diakinesis stage PF oocyte. (*b*) Quantification of fluorescence intensity of each focus in DAPI-stained nuclei in PF oocytes. Six or five fluorescence intensity values from one nucleus were sorted in ascending order and normalized to the third strongest value (*n* = 19 and *n* = 13 for five and six, respectively). In the nuclei with five condensed chromosomes, the highest fluorescence intensities were two to four times higher than the others. (*c*) Chromosome staining by multiplexed FISH. The top and bottom panels represent cases with five and six condensed chromosomes (CCs), respectively. Chromosomes 1, 2 and X1 were probed with Alexa488, Cy3 and Alexa647, respectively. Chromosome X2 was labelled with a combination of Alexa488 and Cy3. Images of the chromosome territories are shown in the right panels. Scale bars, 5 µm.

DAPI microscopy of *S. ratti* PF oocytes revealed five or six CC in each cell (figure 6*a*). The percentage of five CC cases in the oocytes varied from 0% to 67% depending on the worm (electronic supplementary material, figure S3). In oocytes with five CCs, one CC showed higher fluorescence intensity than the others, whereas no such difference was observed for six CC nuclei (figure 6*b*). Multiplexed FISH was then performed to differentiate each chromosome. The results showed that in oocytes with six CCs, all chromosomes or chromosome regions (Chr1, 2, X1, X2) were successfully identified by the FISH and all chromosomes were observed as pairs, representing 2*n* = 6 (figure 6*c*). In oocytes with five CCs, while Chr1 and Chr2 were present as pairs, chromosome X was present as a single with higher fluorescence intensity, probably representing a conjoined form of two homologous X chromosomes. On closer inspection, a pair of the ChrX1 subcluster region in chromosome X was particularly clustered together in the nuclei. Taken together, these results suggest that PF produce L1 larvae with two different karyotypes (probably XX/XO) and that bivalent formation of X chromosomes in PF oocytes may be associated with the XO male generation.

## 4. Discussion

Fragmented genome assemblies hinder in-depth understanding of chromosome evolution [43]. In this study, we successfully obtained chromosome-level genome assemblies for two *Strongyloides* species and the outgroup species *R. diutinus* using a long-read sequencing and Hi-C. Frequent intra-chromosomal rearrangements in the *Strongyloides* clade have previously been suggested [7]. With the new chromosome-level assemblies, we have confirmed this and further obtained a much clearer view of chromosome rearrangements and fusions. The results of the new assembly clearly show that intra-chromosomal rearrangements are very frequent in the *Strongyloides* clade compared to other nematode groups such as the genera *Caenorhabditis* [44], *Pristionchus* [40] and *Bursaphelenchus* [11], which somewhat maintain synteny blocks between distantly related species, although comparisons have not been made quantitatively. Another genomic feature of this group is likely to be occasional chromosome fusions. *S. venezuelensis* has two chromosomes, and we found that one of them is likely the result of fusion events of multiple ancestral chromosomes syntenic to *S. ratti* chromosomes I and X. Chromosome fusion has been also reported in *S. papillosus* [22], which is closely related to *S. venezuelensis*. *S. papillosus* undergoes chromatin diminution where the centre of a fused chromosome corresponding to *S. ratti* ChrX is removed by programmed DNA elimination during mitosis, the process that produces XO males [22]. In contrast, chromatin reduction or male production has not been reported in *S. venezuelensis.* [28]*.* Therefore, comparing the chromosomes of these two species can be a good model to study the mechanisms underlying chromatin reduction and male production.

We identified a sixth chromosome in *R. diutinus*, which was not found in the previous cytological observation [7]. The sixth chromosome is smaller (2 Mb) than the others (6.5–11.2 Mb). Interestingly, although the Hi-C contact map identified six distinct clusters and the DAPI observation of the eggs always identified six chromosomes, the focus of the sixth chromosome in the oocytes was not very clear; it was observed to be smaller than expected from the scaffold size or even undetectable in some cells. Therefore, it remains to be determined whether the sixth chromosome is a normal chromosome or a special chromosome that shows abnormal behaviour at certain stages of development.

As previously reported [45], our results also suggest that the X chromosome (ChrX1 and ChrX2) of *S. ratti* is functionally involved in sex determination. Long-term conservation of sex chromosomes has been suggested in a variety of organisms [42]. The conserved synteny in the X chromosome in the *Strongyloides* clade, in contrast to autosomal fusions and rearrangements, suggests that the X chromosome has been functional from *Rhabditophanes* to *Strongyloides*. Single worm sequencing analysis showed that *S. ratti* males and females have different amounts of X chromosome. In XO males, half the depth to autosomes was expected in one sex chromosome, but ChrX of adult *S. ratti* males showed less than half. In addition, adult females (both PF and FLF) showed slightly less depth in ChrX. This may be explained by autosome-biased amplification in the giant nuclei of adult worms [46]. This may also contribute to differences in X-chromosome abundance between the sexes, but we showed that L1 larvae from PF eggs, which do not have giant nuclei, possess two types of X-chromosome abundance, possibly corresponding to male and female. Furthermore, our cytological observation revealed that two homologous X chromosomes can be joined to form a bivalent chromosome in oocyte germ cells, which leads to the assumption that one of the X chromosomes is eliminated during the early period of embryogenesis and aggregation of two homologous X chromosomes may be one of the processes of elimination. The subclusters observed in the *S. ratti* Hi-C contact map at the X chromosome region are also possibly important for chromosome elimination, as the ChrX1 region appears to act as a hinge to link the two chromosomes.

In this study, we revealed syntenic relationships of chromosomes in the *Strongyloides clade* and sex chromosome dynamics in the life cycle of *S. ratti*. As the new chromosome-level assemblies for three species provided a better understanding of genome evolution, generating the same level of assemblies for other key species, including *P. trichosuri* and *S. papillosus*, would be necessary for a deeper understanding of chromosome evolution in the *Strongyloides* clade. In addition, live imaging of chromosome dynamics using transgenic nematodes expressing fluorescent nuclear proteins will be very useful to understand the mechanisms of XO male generation during mitotic parthenogenesis.

## Data Availability

All the data, including genome assemblies and raw reads for *S. ratti*, *S. venezuelensis* and *R. diutinus*, are available from BioProject (accession number PRJEB65029): https://www.ncbi.nlm.nih.gov/bioproject/?term=PRJEB65029. [47] The data are provided in electronic supplementary material [48].
